# Characterizing rescue performance in a tertiary care medical center: a systems approach to provide management decision support

**DOI:** 10.1186/s12913-021-06855-w

**Published:** 2021-08-20

**Authors:** Susan P. McGrath, Todd MacKenzie, Irina Perreard, George Blike

**Affiliations:** 1 Analytics Institute, Dartmouth-Hitchcock Health, Lebanon, NH 03756 USA; 2 Department of Biomedical Data Science, Dartmouth College, Hanover, NH 03755 USA; 3 Department of Anesthesiology, Dartmouth-Hitchcock Health, Lebanon, NH 03756 USA

**Keywords:** Patient deterioration, Patient safety, Rescue system, Healthcare systems

## Abstract

**Background:**

Allocation of limited resources to improve quality, patient safety, and outcomes is a decision-making challenge health care leaders face every day. While much valuable health care management research has concentrated on administrative data analysis, this approach often falls short of providing actionable information essential for effective management of specific system implementations and complex systems. This comprehensive performance analysis of a hospital-wide system illustrates application of various analysis approaches to support understanding specific system behaviors and identify leverage points for improvement. The study focuses on performance of a hospital rescue system supporting early recognition and response to patient deterioration, which is essential to reduce preventable inpatient deaths.

**Methods:**

Retrospective analysis of tertiary care hospital inpatient and rescue data was conducted using a systems analysis approach to characterize: patient demographics; rescue activation types and locations; temporal patterns of activation; and associations of patient factors, including complications, with post-rescue care disposition and outcomes.

**Results:**

Increases in bedside consultations (20% per year) were found with increased rescue activations during periods of resource limitations and changes (e.g., shift changes, weekends). Cardiac arrest, respiratory failure, and sepsis complications present the highest risk for rescue and death. Distributions of incidence of rescue and death by day of patient stay may suggest opportunities for earlier recognition.

**Conclusions:**

Specific findings highlight the potential of using rescue-related risk and targeted resource deployment strategies to improve early detection of deterioration. The approach and methods applied can be used by other institutions to understand performance and allow rational incremental improvements to complex care delivery systems.

**Supplementary Information:**

The online version contains supplementary material available at 10.1186/s12913-021-06855-w.

## Background

Death due to medical error has been cited as the third leading cause of death in the U.S. [1]. While several decades of patient safety work focused on reducing deaths by attempting to prevent conditions that cause deterioration, there is growing recognition that high and low mortality hospitals are distinguished less by complication rates than by how successfully they recognize and manage serious but treatable problems once they occur [2]. A number of interventions have been designed and employed to address preventable deaths, known as failure to rescue (FTR) events [3], including: rapid response and code teams to provide prompt response to patient deterioration [4]; algorithms to estimate patient state or risk of death [5, 6]; and continuous patient monitoring to support early recognition of deterioration [7, 8]. Several multi-center studies have examined the association of interventions and other hospital [9], and patient [3] characteristics with FTR event incidence and mitigation. Outcome [10–12], activation criteria [13], and temporal aspects of response team utilization [14], have also been studied.

Despite high adoption rates of tactics associated with FTR event reduction, a greater than two-fold variation in hospital mortality associated with serious but treatable events still exists [2], suggesting additional opportunity for improvement [15, 16]. One contributor to this situation may be the paucity of hospital-specific system performance analysis of rescue care activity in available studies [1]. While multi-center studies relying on administrative data may provide epidemiological evidence of *which* hospital-level features are associated with FTR (e.g., high nursing ratios associated with lower code rates), they provide little insight into *how* such systems can be effectively implemented. Studies using engineering systems design and improvement methodologies [17–19] demonstrated the importance of understanding performance of existing systems to identify system leverage points and specific improvement opportunities. Thus, the adoption of high-level rescue care tactics without understanding institution-specific rescue system performance (e.g., by characterizing structure, process and outcome metrics) may explain the wide variation in intervention effectiveness and overall rescue outcomes reported [20].

This observational retrospective study evaluates rescue care system performance in a single tertiary care hospital with the primary aim of identifying system leverage points for improvement. The analysis approach and methods stem from systems engineering and improvement domains, which are exploratory in nature, proceeding from general performance analysis to leverage point identification and prioritization of opportunities for improvement. The metrics selected for study were based initially on existing evidence in the literature, previous system-level analysis at the study institution [21], and commonly reported rescue measures, and also influenced by availability of data. Impact of rescue system components, i.e., types of rescue responses, were of particular interest as these are obvious system leverage points. The analysis described here includes adult patients treated over a four-year interval and details associations of: demographics and characteristics of rescued patients; locations of care and complications (defined per the practice in FTR literature) present; rescue intervention types and utilization; temporal patterns of rescue activation; and rescue outcomes of death, survival, and care escalation. Results provide understanding of how the current system and its primary components behave and highlight gaps in performance which can aid in identification of tactics to improve early recognition and response of deteriorating patients. While the specific findings may not apply to all healthcare institutions, especially those with significantly different rescue system designs, the methods of analysis are generalizable and illustrate the importance of employing an engineering analysis approach to healthcare systems improvement.

## Methods

### Setting

The study institution is an academic medical center in New England with Level 1 trauma center designation, a comprehensive children’s hospital, cancer center, more than 1500 primary and specialty care providers in nearly every specialty area, and more than 10,000 employees. Annually, the institution has more than 1.8 million outpatient visits, over 30,000 emergency room visits, nearly 50,000 discharges, and performs more than 20,000 procedures. Patient acuity is in the top 5% in the United States, and the rural nature of the areas served present a particular challenge in terms of both transportation and weather [22].

### Rescue system

The rescue system is a core component of the life safety program at the study institution supported by policies and procedures defining rescue hierarchy, activation guidelines, and interventions. The system, in place and stable since 2009, is based on tiered response for managing events and includes: life safety consults (LSCs) with dedicated critical care nurses; rapid response activations (RRT known locally as HERT); stat airway; and code blue [see in Additional file 1 table, which includes descriptions of each response type, roles, and activation criteria]. The rescue system also includes monitoring of all inpatients, including continuous pulse oximetry monitoring with threshold-activated bedside alarms and alarm escalation in the general care setting. The organization has a committee that performs review of all mortality cases and feedback from these reviews as well as rescue system performance data are shared at the hospital unit level monthly.

### Dataset

Hospital operational data including patient days, bed counts, bed occupancy, and rescue activity types and inpatient medical records were obtained for four consecutive fiscal years (July 2011–June 2015). This retrospective study was performed with Institutional Review Board approval. Patient data obtained from the electronic medical record using industry standard queries included age, gender, relationship status, complications, disposition at discharge, medical diagnostic codes (MDC), and MDC type (medical or surgical). Complications associated with existing measures of FTR [23, 24], identified by secondary, non-present on admission ICD-9 codes, were gathered including: respiratory failure, pneumonia, stroke, sepsis, acute kidney injury, shock cardiac arrest, gastrointestinal bleed/ulcer, deep vein thrombosis, and hemorrhage. Rescue event data, including type, date, location, and post-rescue patient disposition were obtained from an institutional database. Excluded from analysis were patients under the age of 18 and patients with incomplete data. Matlab® (Mathworks®, Natick, MA, version 2016a) was used to integrate rescue and patient data and facilitate analysis, while R (R Foundation for Statistical Computing, version 3.3.1) was employed for statistical analyses.

### Hospital and patient demographics

Descriptive statistics summarized patient age, gender, race, and relationship status. Patient days were calculated using midnight census, while length of stay (LOS) represents the difference between admission and discharge time. Bed counts and percent occupancy were calculated using institutional standards. Patient location at time of rescue was classified as critical care (including cardiovascular critical care), progressive care (also known as intermediate care), or surgical or medicine general care (designated by the hospital based on typical majority patient population). Mortality was determined as percentage of all patients discharged deceased.

### Rescue care activation utilization

Rescue care activation counts were stratified by rescue type and inpatient unit. Temporal trends in rescue incidence were examined using a Poisson model with offset specified as logarithm of patient days. Linear regression was applied to assess relationships between rescue incidence and mortality using quarterly rates. Given that emergency intubations in critical care or during surgery are not included in the database and the infrequent occurrence of stat airway activities in other settings (*n* = 79), stat airways were excluded from subsequent analysis.

### Rescue care activity outcomes

Association of rescue with LOS and mortality was examined by comparing frequency of discharge and mortality by day since admission between patients with and without rescue. Three outcomes representing the disposition of patients immediately after rescue events were examined: patient deceased; transferred to higher/specialized level of care; or remained in the same level of care. Patient disposition was stratified by rescue type, segmented into medical and surgical units using methods for proportions.

### Rescue care activation temporal patterns

Incidence of rescue in relation to day of hospital stay was calculated as the number of patients who had a rescue on a particular day of their stay divided by the total number of patients with a LOS at least as long as the day in question. Daily probability of rescue was calculated for each day of patient stay for general care surgical and medicine patients. The role of time of day, day of week, and month of year on frequency of rescue was examined. The effect of hour of activation was analyzed using methods for binomial proportions with a smoother. Impact of day of week and month of year on the rate of rescue was assessed using Poisson regression with offset for patient volume. Temporal data were segmented by type of rescue activity.

### Patient characteristics and outcomes associated with rescue

Patient age was bracketed into six groups of 10 years with the exception of the youngest and oldest patients, where 18–39 and > 80 years old were used. For analyses involving gender, age, relationship status, and MDC type, one group was selected from each category as the reference group, i.e., under 40 years old, male, single, and surgical. For analyses involving MDC category and complications, data from patients with the MDC or complication were used in comparison to all patients with other MDCs or without the complication.

Adjusted hazard ratios obtained from Cox’s multivariable proportional hazards model are reported to understand the ability of each patient characteristic to predict death at discharge, rescue, and rescue type. A chi-square analysis of coefficients was used to understand the ability of a characteristic to differentially predict types of rescue (LSC, Code, HERT) and death or transfer across types of rescue. Among patients with rescues, methods for binary outcomes, including Pearson chi-square tests and multivariable logistic regression, were used to evaluate the ability of patient characteristics to predict immediate rescue outcomes of death or transfer to a higher care level. Odds ratios adjusted for age, sex, patient type (medical or surgical) and marital status are reported.

## Results

### Hospital and patient demographics

Over 67,000 patients met study inclusion criteria with a 5.6 day (SD = 8.1) average LOS. Overall mortality rate was 2.73% with a decline in the annual rate of 0.27% (*p* = 0.06). A total of 4003 rescue activations were documented. LSCs represented 77% of all rescue activities followed by HERT (*n* = 520), code blue (*n* = 322) and stat airway (*n* = 79) activations. For a full summary of patient characteristics and hospital operational metrics see Additional file 2 table.

### Rescue care activation utilization

Table 1 shows utilization of rescue care by type. Across all care settings, there were 9.2 LSCs per 1000 patient days, over 5 times higher than HERT and 8 times higher than code blue activations. Comparison across care unit types demonstrated more frequent consults in surgical versus medicine units (9.0 vs 6.4 per 1000 patient days). The number of consults in progressive care units was substantially higher at 22.8 per 1000 patient days, whereas in critical care it was lower (0.2 per 1000 patient days). A significant overall increase in LSCs was observed (20% per year, *p* < .001) with significant increases in surgical (10%, *p* = 0.002) and medicine units (24%, *p* < .001). Linear regression analysis comparing incidence of LSCs to mortality rates showed the effect of a 1% relative increase in LSCs to be a 0.8% decrease in mortality, or 8 less deaths per 1000 discharges.

**Table 1:** Rescue frequency stratified by rescue activity types. Rates of each rescue activity were calculated and normalized by 1000 patient days. The percentage change per year over the study period with 95% CI and *p*-value are included. Counts, percentages of total events in each patient unit type and standard errors are listed

Event type	#/1000 Patient days	% change per year over study period, (CI), p	Unit type	#/1000 Patient days	% change per year over study period, (95% CI), p	Patient Disposition after Rescue^**c**^	% (Std. error)
LSC^a^	9.24	20% (16–23%), < 0.0001	Critical Care Units	0.16	− 14% (− 54–60%), 0.6	Stayed in room	75 (0.3)
						Transfer	25 (0.2)
						Died	0(−)
			Progressive Care Units	22.8	5% (−1–12%), 0.07	Stayed in room	82 (0.3)
						Transfer	18 (0.1)
						Died	0(−)
			Surgical Units	8.97	10% (3–16%), 0.002	Stayed in room	75 (1.7)
						Transfer	25 (2.9)
						Died	0(−)
			Medicine Units	6.44	24% (18–31%), < 0.0001	Stayed in room	74 (1.4)
						Transfer	26 (2.3)
						Died	0.1 (2.7)
HERT^b^	1.67	6% (−1–13%), 0.08	Critical Care Units	N/A^d^	N/A	N/A	N/A
			Progressive Care Units	2.82	9% (−8–30%), 0.3	Stayed in room	63 (0.2)
						Transfer	38 (0.2)
						Died	0(−)
			Surgical Units	1.05	−18% (−31- -3%), 0.02	Stayed in room	34 (8)
						Transfer	65 (5.8)
						Died	1 (9.8)
			Medicine Units	1.34	6% (−5–18%), 0.3	Stayed in room	40 (4.5)
						Transfer	58 (3.8)
						Died	2 (5.8)
Code blue	1.14	0.98% (−10–6%), 0.5	Critical Care Units	4.05	0.98% (−13–11%), 0.8	Stayed in room	64 (0.2)
						Transfer	2 (0)
						Died	35(0.2)
			Progressive Care Units	0.54	1.08% (−27–6%), 0.7	Stayed in room	8 (0.1)
						Transfer	83 (0.3)
						Died	1 (0.1)
			Surgical Units	0.23	0.85% (− 41–22%), 0.4	Stayed in room	8 (19.5)
						Transfer	71 (11)
						Died	21 (18.2)
			Medicine Units	0.26	1.1% (−14–39%), 0.5	Stayed in room	9 (13.1)
						Transfer	62 (8.4)
						Died	28 (11.6)

^a^ Life Safety Consult

^b^ Hitchcock Early Response Team

^c^ Patient disposition after rescue for medical and surgical patients in general care is specified. The after-rescue outcomes analyzed were patient stayed in room, patient was transferred to a higher level of care, patient died

^d^ Critical care units act as their own HERT team and therefore no database entry is made for this activation type (see Additional file 1)

Progressive care units had the highest HERT utilization rate at 2.8 per 1000 patient days. HERT activations were similar for medicine and surgical units at 1.34 and 1.05 per 1000 patient days, respectively. There was no difference in overall rate of HERT activations, although surgical HERT activations decreased (− 18%, *p* = .02). Code blue activations were most prevalent in critical care at 4.1 per 1000 patient days. The rate of code blue activations in progressive care units was over twice that in either the medicine or surgical general care settings (0.5 vs 0.3 and 0.2 per 1000 patient days), but well over 7 times less than in critical care. There was no change in rate of code blue activations.

### Rescue care activity outcomes

Table 1 provides comparison of patient disposition for rescue activations. Very few patients were documented in the rescue database as deceased immediately after LSCs (0.1% of all LSCs in medicine units, 0% in surgical units, and higher levels of care). Death rate was higher for HERTs (1 and 2% in medical and surgical general care units, respectively) and code blue activations (21–35% across all care settings). The vast majority of patients who had LSCs stayed in their rooms (74–75%). The reverse was true for patients with HERT activations where 58–65% of patients were transferred. The majority of patients with code blue activations were transferred to higher levels of care (62–83% in progressive and general care settings). There was no difference between medical and surgical units for the three types of patient disposition across rescue activity types.

**Fig. 1:**
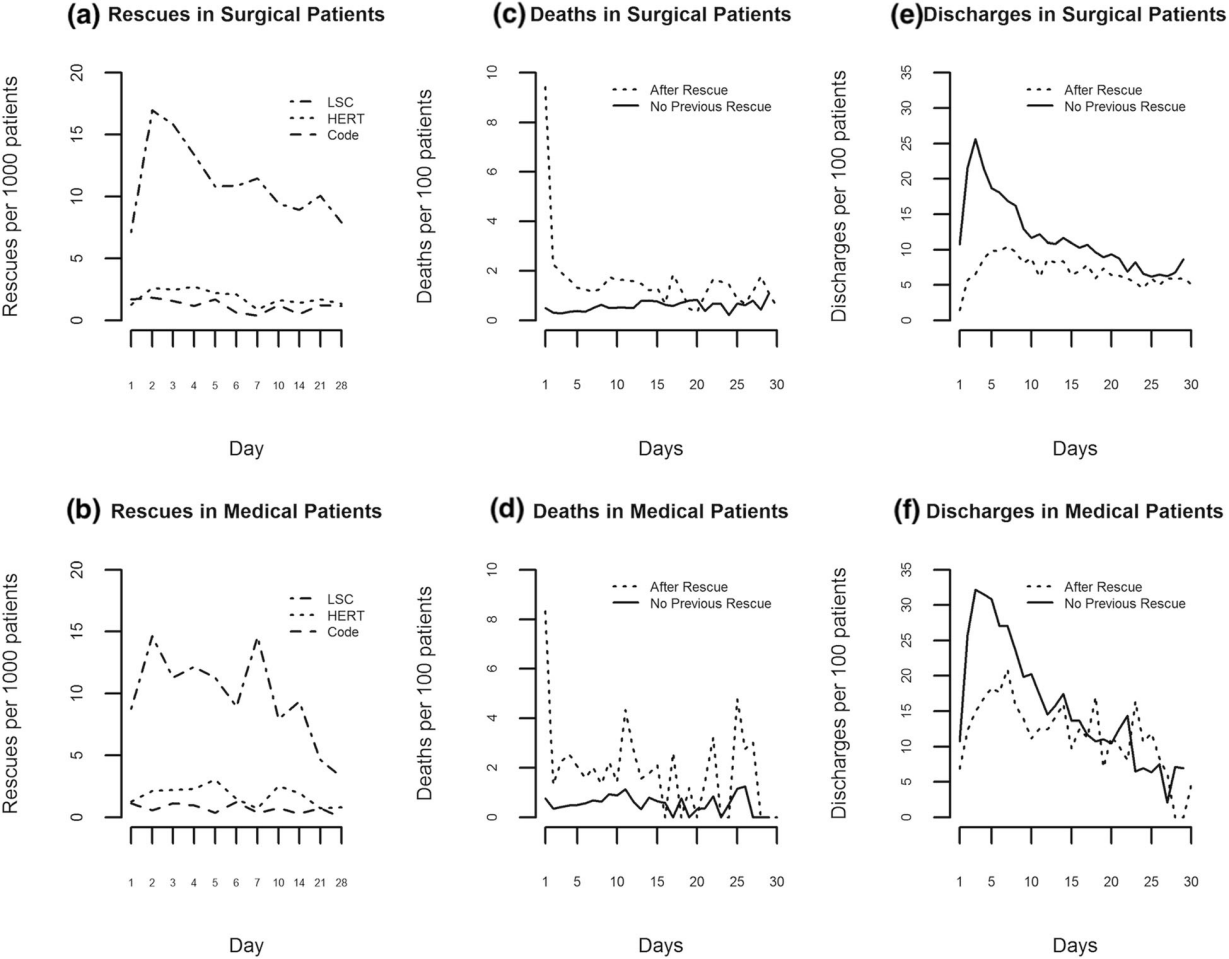
Rescue incidence and outcomes. Rate of rescue by rescue type is shown by day of patient stay in **a** medical and **b** surgical inpatient units. Rate of death at discharge (**c**, **d**) and rate of discharge on each day of patient stay (**e**, **f**) are shown by for each day of patient stay for patients who did and did not receive rescue prior to the day in consideration.

Figure 1 shows incidence of discharge, rescue, and death by day of patient stay for medical and surgical patients, with discharge and death analysis segmented by patients who did or did not receive rescue. There is a marked difference in incidence of discharge between the two groups, with maximum incidence for the no-rescue group on day 3 and on day 7 for the rescue group. Death incidence distributions follow similar trends, with a maximum on stay day 1 for rescued patients.

### Rescue care activation temporal patterns

**Fig. 2:**
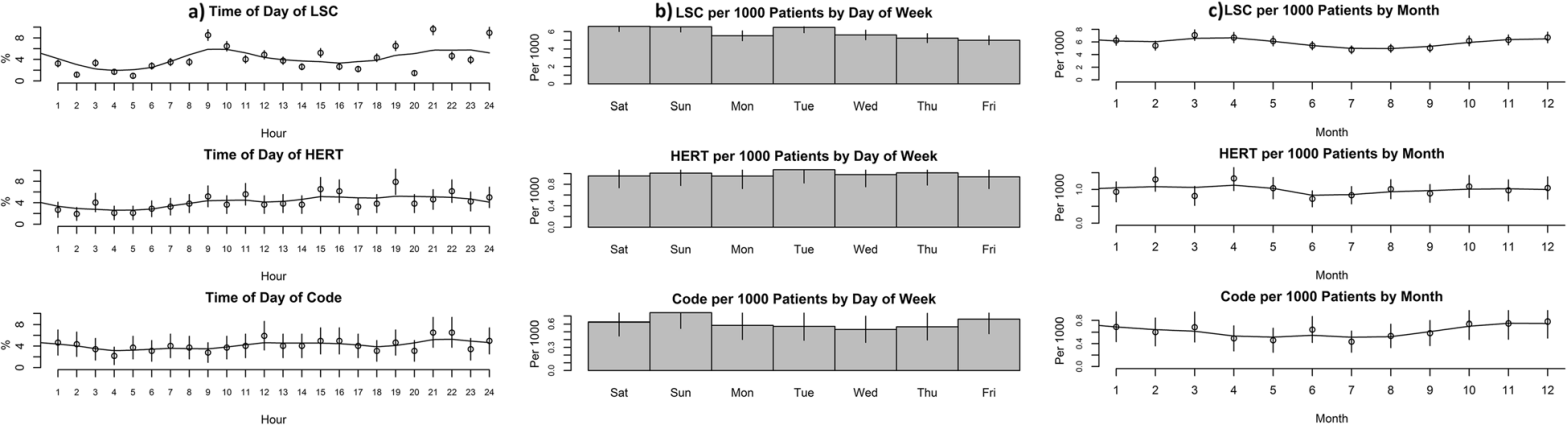
Temporal analysis of rescue activations. Percent of total rescue activations are shown in **a** by hour of day for LSC, HERT and Code blue activations. Activations per 1000 patients are shown in **b** by day of week and in **c** by month of year. Mean values and confidence intervals are indicated by circles and vertical lines for each interval.

Normalized rescue incidence by day of patient stay across all care settings and rescue activity types was highest on day 2 (see Fig. 1). Over 35% of all rescues occur within the first 3 days of patient admission, and nearly 55% within the first 5 days. A diurnal pattern for LSCs by hour of day was seen (Fig. 2a), with peaks corresponding to the period just after shift changes. HERT and code blue activations were less frequent in early morning hours, and relatively constant throughout the rest of the day. LSC rate is higher on weekends than other days (*p* < 0.0001) by 18, 95% CI [10, 27%] (Fig. 2b). The rate of LSCs is 21, 95% CI [9, 35%] higher on Tuesdays than other weekdays (*p* = 0.0003). Analysis showed no difference in the rate of HERTs or code blue activations between weekend and weekdays, or Tuesdays vs other days (*p* > 0.1). LSCs occurred at a lower rate June through September than other months (*p* = 0.0004) (Fig. 2c). There was no significant variation in HERT activations although frequency was slightly lower in summer months. Code rates varied cyclically over the year (*p* = 0.0002 for test of sinusoidal variation) with the nadir occurring during summer months.

### Patient characteristics outcomes associated with rescue

Table 2 provides a summary of patient characteristics associated with death at discharge and rescue. Gender analysis revealed no difference in risk of death for males compared to females, although males were 21% (*p* < .00001) more likely to receive rescue. Age analysis showed monotonically increasing risk for death over age 40. Patients over 70 were more than 8 times more likely to die as compared to patients under 40. The same trends were seen in hazard ratios for rescue, although differences between age groups were not as marked. Single patients were more likely to die or have rescue than patients in other relationship categories. Surgical patients were at lower risk for death than medicine patients, but presented higher risk of rescue, especially for codes (hazard ratio 2.63 *p* < .001).

**Table 2:** Impact of patient characteristics on death at discharge and rescue. Hazard ratios for death at discharge and rescue obtained from Cox’s proportional hazards model were calculated for each feature within a category using reference characteristics. Hazard ratios, adjusted for controlling for age, sex, marital and surgical/non-surgical patient status, are presented along with confidence intervals and *p* values. Only the five MDC-DRGs with the highest hazard ratios are shown

**Category**	**Patient Characteristic**		**Death**		**Rescue**	
			**Hazard Ratio (95% CI)** **controlling for age, sex, marital and surgical**	**p**	**Hazard Ratio (95% CI)** **controlling for age, sex, marital and surgical**	**p**
Demographics (reference: Female, Age < 40, Relationship status: Single)	Male		1.08 (0.98,1.18)	0.10	1.21 (1.13,1.3)	0.00
	Age 40–50		2.06 (1.55,2.75)	0.00	1.93 (1.64,2.27)	0.00
	Age 50–60		2.92 (2.27,3.75)	0.00	2.16 (1.87,2.49)	0.00
	Age 60–70		4.02 (3.15,5.13)	0.00	2.51 (2.18,2.88)	0.00
	Age 70–80		5.26 (4.12,6.73)	0.00	3.12 (2.71,3.59)	0.00
	Age > 80		8.4 (6.56,10.76)	0.00	3.16 (2.71,3.69)	0.00
	Relationship status: Couple		0.8 (0.7,0.92)	0.00	0.67 (0.61,0.74)	0.00
	Relationship status: Divorced or separated		0.81 (0.69,0.95)	0.01	0.92 (0.82,1.03)	0.14
	Relationship status: Widowed		0.9 (0.76,1.06)	0.20	0.85 (0.74,0.96)	0.01
	Relationship status: Unknown		0.98 (0.77,1.26)	0.90	0.65 (0.53,0.8)	0.00
MDC Type (reference: Nonsurgical patient)	Surgical patient		0.88 (0.8,0.97)	0.01	1.71 (1.6,1.84)	0.00
Complications (reference: no complication)	Shock / Cardiac Arrest		6.81 (6.03,7.69)	0.00	6.44 (5.72,7.24)	0.00
	Respiratory Failure		6.4 (5.68,7.22)	0.00	7.49 (6.9,8.13)	0.00
	Sepsis		3.53 (3.14,3.98)	0.00	5.96 (5.42,6.55)	0.00
	AKI		2.9 (2.59,3.24)	0.00	4.58 (4.21,4.99)	0.00
	Pneumonia		2.37 (2.1,2.67)	0.00	6.53 (5.97,7.13)	0.00
	Stroke		1.92 (1.62,2.27)	0.00	2.13 (1.83,2.49)	0.00
	GI Bleed / Ulcer		1.61 (1.33,1.95)	0.00	3.43 (2.97,3.97)	0.00
	DVT		0.74 (0.57,0.98)	0.03	3.39 (2.86,4.03)	0.00
	Hemorrhage		1.01 (0.87,1.17)	0.92	1.35 (1.21,1.52)	0.00
**MDC-DRG Category** (reference: all other categories)	**Death**			**Rescue**		
	**MDC category**	**Hazard Ratio (Lo, Hi)**	**p**	**MDC category**	**Hazard Ratio (Lo, Hi)**	**p**
	Infectious and Parasitic diseases	2.71 (2.41,3.05)	0.00	Multiple Significant Trauma	3 (2.44,3.68)	0.00
	Factors influencing health status and contact with health services	1.99 (1.27,3.12)	0.00	Human immunodeficiency virus infections	2.8 (1.24,6.35)	0.01
	Multiple Significant Trauma	1.9 (1.44,2.52)	0.00	Infectious and Parasitic diseases	2.68 (2.4,2.99)	0.00
	Diseases & Disorders of the Respiratory System	1.84 (1.64,2.07)	0.00	Diseases & Disorders of the Respiratory System	2.61 (2.38,2.86)	0.00
	Diseases and disorders of the nervous system	1.38 (1.23,1.55)	0.00	Substance use and substance-induced organic mental disorders	2.03 (1.34,3.06)	0.00

All complications except hemorrhage were predictors of death. Patients with shock/cardiac arrest or respiratory failure were more than 6 times more likely to die than patients without those complications, while patients with sepsis were over 3 times more likely to die. All complications were predictors of rescue. Respiratory failure patients had 7.5 times risk of rescue than other patients, pneumonia and shock/cardiac arrest each had risk ratios over 6. Patients with several MDCs had risk ratios for death and rescue that were much higher than others (e.g., infectious and parasitic diseases: death hazard ratio 2.71, rescue 2.68 *p* < .001).

Considering outcome after rescue across all categories (see Table 3), males were somewhat more likely to die (hazard ratio 1.18 *p* < .001) or be transferred (hazard ratio 1.2 *p* = .0023) after rescue as compared to females. Increasing age amplifies risk of both death and transfer after rescue, although the rate of increase is more significant for death (e.g., hazard ratio 12.38, *p* < .001 for death and 5.0, *p* < .001 for rescue in patients over 80). Most relationship categories had risk of death and transfer significantly lower than patients with single status. Surgical patients are at 50% (*p* < .001) higher risk for death and 2.75 (*p* < .001) times more likely to be transferred than medicine patients. When considering impact of age, gender, relationship status, and MDC type on specific rescue types, age was the only category predictive of death and transfer after LSCs, and patients who declared themselves married had a significantly lower risk than single patients.

**Table 3:** Impact of patient characteristics on patient disposition immediately following a rescue event. Odds ratios were calculated for death and transfers to higher levels of care, controlling for age, sex, relationship and surgical status. Ratios are shown along with confidence intervals and p values. Only the five MDC-DRGs with the highest odds ratios are shown

**CATEGORY (reference categories)**	**Patient feature**	**Predictors of DEATH controlling for age, sex, marital and surgical**			**Predictors of TRANSFER controlling for age, sex, marital and surgical**	
		**Odds Ratio (Lo, Hi)**		**p**	**Odds Ratio (Lo, Hi)**	**p**
**Demographics** (Female, Age < 40, Single)	Male	1.18 (1.08, 1.29)		0.00	1.21 (1.07, 1.38)	0.00
	Age 40–50	2.65 (1.98, 3.54)		0.00	3.12 (2.25, 4.35)	0.00
	Age 50–60	4.01 (3.11, 5.16)		0.00	3.53 (2.61, 4.78)	0.00
	Age 60–70	5.42 (4.24, 6.93)		0.00	3.96 (2.95, 5.33)	0.00
	Age 70–80	7.76 (6.06, 9.93)		0.00	5.43 (4.03, 7.33)	0.00
	Age > 80	12.38 (9.63, 15.9)		0.00	5 (3.62, 6.91)	0.00
	Relationship status: Couple	0.61 (0.53, 0.7)		0.00	0.65 (0.55, 0.78)	0.00
	Relationship status: Divorced or separated	0.76 (0.65, 0.9)		0.00	0.87 (0.7, 1.08)	0.19
	Relationship status: Widowed	0.75 (0.63, 0.89)		0.00	0.74 (0.58, 0.95)	0.02
	Relationship status: Unknown	0.84 (0.65, 1.09)		0.17	0.61 (0.42, 0.9)	0.01
**Surgical** (nonsurgical patient)	Surgical patient	1.51 (1.37, 1.65)		0.00	2.74 (2.37, 3.17)	0.00
**Complications** (no complication)	Shock/Cardiac Arrest	27.46 (23.57, 31.99)		0.00	7.17 (5.83, 8.81)	0.00
	Respiratory Failure	25.18 (22.25, 28.49)		0.00	14.43 (12.47, 16.7)	0.00
	Sepsis	14.62 (12.86, 16.63)		0.00	9.72 (8.3, 11.4)	0.00
	AKI	8.41 (7.49, 9.46)		0.00	5.66 (4.89, 6.56)	0.00
	Pneumonia	9.6 (8.47, 10.88)		0.00	8.64 (7.41, 10.8)	0.00
	Stroke	3.5 (2.91, 4.2)		0.00	2.41 (1.86, 3.13)	0.00
	GI Bleed / Ulcer	3.75 (3.05, 4.6)		0.00	3.84 (3, 4.92)	0.00
	DVT	2.32 (1.74, 3.09)		0.00	4.5 (3.41, 5.93)	0.00
	Hemorrhage	1.55 (1.32, 1.82)		0.00	1.7 (1.4, 2.07)	0.00
**MDC-DRG** (all other categories)	**Death after Rescue**			**Transfer after Rescue**		
	**MDC**	**Odds Ratio (Lo, Hi)**	**p**	**MDC**	**Odds Ratio (Lo, Hi)**	**p**
	Infectious and Parasitic diseases	5.45 (4.81, 6.19)	0.00	Infectious and Parasitic diseases	2.89 (2.36, 3.54)	0.00
	Human immunodeficiency virus infections	4.6 (1.58, 13.3)	0.00	Diseases & Disorders of the Respiratory System	2.68 (2.26, 3.19)	0.00
	Multiple Significant Trauma	3.15 (2.34, 4.24)	0.00	Injuries, Poisonings & Toxic Effects of Drugs	1.88 (1.32, 2.66)	0.00
	Diseases & Disorders of the Respiratory System	2.02 (1.26, 3.22)	0.00	Diseases & Disorders of the Hepatobiliary System & Pancreas	1.59 (1.22, 2.07)	0.00
	Factors influencing health status and contact with health services	1.57 (1.39, 1.76)	0.00	Myeloproliferative Diseases & Disorders, Poorly Differentiated Neoplasms	1.5 (1.1, 2.07)	0.01

All complications were predictors of death after rescue, with patients having shock/cardiac arrest and respiratory failure each having risks more than 25 times higher than other patients. All complications were also predictors of transfer after rescue, although not to the same extent, with respiratory failure and sepsis having the highest risk (odds ratios 14.43 and 9.72, respectively, *p* < .001). Several complications were predictive of death and transfer for specific rescue types. Patients with hemorrhage, respiratory failure, and sepsis were 3.6, 3.4 and 2.24 times more likely to die after HERT as compared to patients without those conditions, respectively. Shock/cardiac arrest was the only complication with higher risk of death after code at 2.7 times than patients without the complication. Similar patterns emerged for predictors of transfers with respiratory failure and sepsis patients most likely to be transferred after LSC and HERT, and shock/cardiac arrest also significant after a HERT. Patients with respiratory failure were nearly 5 times more likely to be transferred after HERT, while sepsis patients were 3 times more likely.

For 3 of the 9 complication types, the type of rescue activity was predictive of patient death: hemorrhage, respiratory failure, and DVT. For 4 of the 9 complications, the type of rescue received was predictive of transfer: respiratory failure, shock/cardiac arrest, AKI, sepsis and GI bleed/ulcer. MDCs for infectious and parasitic diseases (odds ratio 5.45 *p* < .001), HIV infections (risk ratio 4.6 *p* < .001), significant trauma (odds ratio 3.15 *p* < .001) and respiratory diseases (odds ratio 2.60 *p* < .001) were most predictive of death after rescue. For transfers after rescue, MDCs for infectious and parasitic diseases (odds ratio 2.89 *p* < .001) and respiratory diseases (odds ratio 2.68 *p* < .001) were most predictive. Diseases and disorders of the hepatobiliary system and pancreas and myeloproliferative disorders and poorly differentiated neoplasms were predictive of death after both LSC and HERT. Only risk for diseases and disorders of the nervous system was significant for transfer after code, with a risk two thirds lower than all other MDCs.

## Discussion

The findings of this rescue system assessment provide understanding of current system behavior and highlight effective aspects of the rescue system as well as opportunities for improvement. A significant increase (20% per year) in use of LSCs by general care nurses was observed. LSC increases were associated with a 0.8% decline in mortality (*p* = 0.06). Rates of other rescue activities remained constant and are lower than reported elsewhere [25]. The study institution encourages use of proactive critical care consultation using low-threshold activation criteria of “clinician concern” to enable early recognition of complications and/or general patient deterioration. One might expect successful diagnosis and treatment hours or days earlier in the evolution of a complication prevents sequelae and reduces the need for urgent rescue or resuscitation. Other factors that may impact early recognition via LSCs include: unit staffing levels and clinical experience; increased knowledge of rescue team functions; ubiquitous availability of continuous monitoring data [26, 27]; and cultural barriers to provider communication and RRT activation [28, 29].

Temporal analysis provided useful insights regarding impact of rescue activity and resource availability. Patients are at higher risk of having rescue earlier in their hospitalization, and if a patient had a rescue event, their risk of death and LOS both increased. Additionally, patterns of variation in rescue activity were associated with resource limitations and/or changes (i.e., increased rescue calls after shift changes when new teams assume responsibility for patients and may note issues, and fewer codes during the summer months when supervision and other support resources are typically increased due to staff onboarding). These results expand on previous RRT studies, for example [30] reported an increase in RRT activations during daytime hours vs. nighttime hours.

The difference between peak of death incidence on day 1 of patient stay as compared to peak rate of all rescues that occurred later during patient stays (Fig. 2) may reflect patient-related factors such as acuity on admission or care choices, but might also represent additional opportunity to improve deterioration recognition at the bedside. This observation is consistent with other studies that highlight the need to improve recognition of patient deterioration in addition to developing rescue-related interventions [15, 31], although the current study provides temporal specificity not described elsewhere.

Patient-focused analysis provided understanding of population-specific patterns associated with rescue. The finding that single patients were more likely to die or have rescue than patients in other relationship categories supports existing findings [32]. Higher risk of rescue of surgical and male patients and higher risk of death among medical patients may not be surprising results given known trends and patient characteristics/interventions, but could suggest there is utility in exploring population specific monitoring strategies. Increasing risk of rescue and death with age is expected, however understanding the magnitude and rate of increase provides further context for assessment and can be combined with other risk information, such as gender and relationship status, to formulate population-specific risk profiles. This tactic can be extended to patterns of risk associated with complications and disease categories as well. At the study institution this was accomplished by establishing a committee to review rates of complications and outcomes and charge individual workgroups with developing complication/condition-specific treatment protocols and rescue responses for prioritized condition including sepsis, respiratory failure and DVTs. Given that patient populations and procedures vary across hospitals, an institution-specific approach can provide value through efficient and appropriate allocation of limited resources and clinician attention.

Leverage points for system improvement suggested by the utilization and risk data presented and discussed here include resource allocation (e.g., risk-based patient placement and deployment of consultative rescue resources and staffing/team models based on rescue patterns) and clinical education regarding use of patient risk profiles and associated patient assessment to support earlier detection of deterioration and complications, especially given the growing consensus that failure to detect a complication needing treatment is a primary driver of FTR mortality [2, 33]. Study data also represent baseline performance against which the impact of such interventions can be assessed. More details related to FTR event mitigation and rescue system intervention design and measurement based in part on this work can be found elsewhere [21, 34].

This study is not without limitations. This was a single institution study based in a rural region with little racial diversity, and consequently variation in some aspects of the study population is not representative of hospitals in other regions. Other potentially differentiating factors include a mature life safety program with over 10 years of policies, procedures and management systems in place, critical care nurses available for LSCs at all times, and application of continuous surveillance monitoring as the standard of care for all general care inpatients. Another limitation of this study relates to the rescue database from which data were gathered for analysis; data are entered manually by staff and not formally validated; hence some level of human error and omissions are possible. There is also a degree of flexibility in patient placement in general care units, i.e., every patient in a unit designated as surgical or medical may not have an MDC of that type. This variability may affect comparisons between these unit types, although one could argue that cultural and resource allocation differences that are common across units of similar designation also impact care irrelgarless of variation in patient population.

## Conclusions

This work represents a comprehensive system-level performance analysis of an existing rescue system, with analysis that revealed patterns in activation utilization and impact of patient characteristics on rescue activation and outcomes. The study builds upon previous work highlighting that early recognition of changes in patient state is a key factor in preventing FTR events and underscores the importance of bringing resources and tools (e.g., continuous monitoring and LSCs) to the bedside to enhance early recognition. While results may not be generalizable to other hospitals with different rescue systems, the approach and methods applied can be used by other institutions to understand performance and allow rational incremental improvements to this complex care delivery system.

## Supplementary Information

**Additional file 1.** Rescue activities and teams. Description of the activity, activation criteria, roles and settings for each of the four established rescue activities are provided. The Life Safety Registered Nurse is a Critical Care RN, certified in Advanced Cardiac Life Support and Pediatric Advanced Life Support (ACLS/ PALS), trained specifically as a lead member of the emergency response teams.

**Additional file 2.** Summary of hospital and patient characteristics. General patient characteristics obtained from the medical record were calculated across the 4 years of study data. Hospital data including patient days, count of bed types, bed occupancy and rescue activity types are summarized over the same time period.

## Data Availability

The dataset used for this study is not publicly available due to institutional guidance related to rescue data. Inquiries can be forwarded to Susan McGrath, PhD. At susan.p.mcgrath@hitchcock.org.
