# Supplementary material for: Characterizing rescue performance in a tertiary care medical center: a systems approach to provide management decision support
Source: BMC Health Serv Res. 2021 Aug 20;21:843. doi: 10.1186/s12913-021-06855-w (PMC8379722; doi:10.1186/s12913-021-06855-w)
Supplement: Supplementary file 1 — Additional file 1. Rescue activities and teams. Description of the activity, activation criteria, roles and settings for each of the four established rescue activities are provided. The Life Safety Registered Nurse is a Critical Care RN, certified in Advanced Cardiac Life Support and Pediatric Advanced Life Support (ACLS/ PALS), trained specifically as a lead member of the emergency response teams. [file 12913_2021_6855_MOESM1_ESM.docx]

**Characterizing Rescue Performance in a Tertiary Care Medical Center: A Systems Approach to Provide Management Decision Support**

**Authors**

Susan P. McGrath, Ph.D.

Analytics Institute, Dartmouth-Hitchcock Health, Lebanon, NH 03756 USA

Corresponding author

Email: Susan.p.mcgrath@hitchcock.org

Todd MacKenzie, Ph.D.

Department of Biomedical Data Science, Dartmouth College, Hanover, NH 03755 USA

Irina Perreard, Ph.D.

Analytics Institute, Dartmouth-Hitchcock Health, Lebanon, NH 03756 USA

George Blike, MD

Department of Anesthesiology, Dartmouth-Hitchcock Health, Lebanon, NH 03756 USA

# Additional File 1. Rescue activities and teams. Description of the activity, activation criteria, roles and settings for each of the four established rescue activities are provided. The Life Safety Registered Nurse is a Critical Care RN, certified in Advanced Cardiac Life Support and Pediatric Advanced Life Support (ACLS/ PALS), trained specifically as a lead member of the emergency response teams.

| **Life Safety Consult** | |
| --- | --- |
| Activation | Bedside nurse concern, provider concern. |
| Activity description | Respond to bedside and assess patient. Discuss interventions with bedside staff and providers as necessary. Arrange for transfer if necessary. |
| Roles | Life Safety Registered Nurse (RN) |
| Settings | General care, progressive care, critical care (rarely) |
| **Rapid Response Team (HERT)** | |
| Activation | Patient with: heart rate > 130 or < 40 beats per minute; systolic blood pressure < 90 mmHg; respiratory rate < 8 or > 30 breaths per minute; SpO2 < 90% with supplemental oxygen; acute mental status changes; difficulty in speaking; threatened airway; or staff member concern about patient. |
| Activity description | Notify all local care team members of activation. Respond to bedside and assess patient. Develop appropriate plan of care, discuss interventions with bedside staff and providers as necessary. Arrange for transfer if necessary. Critical Care staff serve as their own rapid response team. |
| Roles | Critical care RN and Respiratory Care Provider; consult with Critical Care Provider if necessary. |
| Settings | General care, progressive care. |
| **Stat Airway** | |
| Activation | Patient is in acute respiratory compromise and there is a potential need for placement of an artificial airway. |
| Activity description | Urgent or emergent placement of an artificial airway. |
| Roles | Anesthesia provider (resident or Certified Registered Nurse Anesthetist (CRNA)), Anesthesia Attending (Pediatric Stat Airway only), Respiratory Care Provider, House Supervisor, Life Safety RN (for activations outside of critical care only). |
| Settings | General care, progressive care, critical care, operating rooms. |
| **Code Blue** | |
| Activation | Patient found in cardiopulmonary arrest. |
| Activity description | Response to a cardiopulmonary arrest by immediately initiating CPR, activating the internal code blue response, and then activating EMS by calling 911. |
| Roles | Two Providers (one Pediatrics provider and one Primary Care provider), Life Safety RN, Licensed Practical Nurse (LPN) or second RN, Medical Assistants, Facilities Staff Members. |
| Settings | General care, progressive care, critical care. |
