# Supplementary material for: Characterizing rescue performance in a tertiary care medical center: a systems approach to provide management decision support
Source: BMC Health Serv Res. 2021 Aug 20;21:843. doi: 10.1186/s12913-021-06855-w (PMC8379722; doi:10.1186/s12913-021-06855-w)
Supplement: Supplementary file 2 — Additional file 2. Summary of hospital and patient characteristics. General patient characteristics obtained from the medical record were calculated across the 4 years of study data. Hospital data including patient days, count of bed types, bed occupancy and rescue activity types are summarized over the same time period. [file 12913_2021_6855_MOESM2_ESM.docx]

**Characterizing Rescue Performance in a Tertiary Care Medical Center: A Systems Approach to Provide Management Decision Support**

**Authors**

Susan P. McGrath, Ph.D.

Analytics Institute, Dartmouth-Hitchcock Health, Lebanon, NH 03756 USA

Corresponding author

Email: Susan.p.mcgrath@hitchcock.org

Todd MacKenzie, Ph.D.

Department of Biomedical Data Science, Dartmouth College, Hanover, NH 03755 USA

Irina Perreard, Ph.D.

Analytics Institute, Dartmouth-Hitchcock Health, Lebanon, NH 03756 USA

George Blike, MD

Department of Anesthesiology, Dartmouth-Hitchcock Health, Lebanon, NH 03756 USA

Additional File 2. Summary of hospital and patient characteristics. General patient characteristics obtained from the medical record were calculated across the four years of study data. Hospital data including patient days, count of bed types, bed occupancy and rescue activity types are summarized over the same time period.

| **Patient characteristics** | | | | |
| --- | --- | --- | --- | --- |
| Total adult patients, count | 67,142 | | | |
| Age, years mean+/-stdev | 59.09 +/-18.52 | | | |
| Gender, percent | 48% Female, 52% Male | | | |
| Race, percent | 98% White, 2% Other | | | |
| Marital status | 51**%** Married, Civil Union, Life partner  15% Divorced, Separated  18% Single  12% Widowed | | | |
| Mortality, percent | 2.73% | | | |
| LOS, Days, mean+/-stdev | 5.6+/-8.1 | | | |
| Patient days, count | 418,620 | | | |
| **Hospital characteristics by care area** | | | | |
|  | **Surgical General care** | **Medicine General care** | **Progressive care** | **Critical care** |
| Patient days, count | 102,992 | 222,943 | 36,859 | 49,291 |
| Number of beds, count | 83 | 105 | 40(45) | 74 |
| Occupancy, percent | 87% | 88% | 85% | 80% |
| **Rescue events by type** | | | | |
|  | **Life safety consult** | **HERT** | **Code Blue** | **Stat airway** |
| Rescues, count | 3082 | 520 | 322 | 79 |
